# 24-day-old newborn with tinea capitis: A case report

**DOI:** 10.51866/cr.902

**Published:** 2025-09-16

**Authors:** Fa'iza Abdullah, CM Nor Azmi Hani Azmina, Che Abdul Rahim Abdul Rahman

**Affiliations:** 1 MD, Department of Family Medicine, International Islamic University of Malaysia (IIUM), Kuantan, Pahang, Malaysia. E-mail: greeny0201@yahoo.com; 2 MBBS, FRACGP, Department of Family Medicine, International Islamic University of Malaysia (IIUM), Kuantan, Pahang, Malaysia.; 3 MBBS, MRCP, AdvMderm, Department of Dermatology, Hospital Tengku Ampuan Afzan, Kuantan, Pahang, Malaysia.

**Keywords:** Tinea capitis, Infant, Newborn, Antifungal agents

## Abstract

Tinea capitis is a common childhood fungal infection that is extremely rare in newborns, with limited data available. Its clinical presentation may closely resemble that of other dermatological conditions such as seborrheic dermatitis, neonatal lupus erythematosus and congenital syphilis. It is essential to make a definite diagnosis, as tinea capitis treatment requires systemic antifungal therapy. However, successful topical monotherapies have been reported. We report a rare case of neonatal tinea capitis diagnosed in a 24-day-old newborn, presenting with multiple scaly plaques on the scalp, discussing the diagnostic criteria, predisposing factors, appropriate treatment and preventive measures. The patient was treated with topical antifungal therapy, resulting in complete resolution within a week and no recurrence at the 2-year follow-up.

## Introduction

Tinea capitis (TC) is a fungal infection of the scalp. It is common among preschool-aged children but rarely affects infants and is even rarer in the neonatal period.^1^ Since 1990, only 30 cases of neonatal TC have been reported globally.^2^ Various dermatophytes and non-dermatophyte fungi can cause the condition. It should be considered in infants with scaling, alopecic patches, annular erythema, kerion or broken hair on the scalp. Several predisposing factors have been identified, including prematurity, low socioeconomic status, poor hygiene and contact with infected family members or domestic animals such as cats.^3,4^

The mainstay treatment is oral antifungals such as fluconazole, itraconazole, terbinafine and griseofulvin, which have been used in infants, although data on their safety and efficacy in neonates are limited. Topical treatment has been prescribed as initial therapy for neonates with superficial dermatophyte skin infection.^2^ However, topical antifungal therapy is typically insufficient for scalp infections due to poor follicular penetration. This case highlights a significant gap in the existing literature by presenting one of the few reported cases of neonatal TC successfully treated with topical therapy and cured within 1 week.^2^ Hence, this case report presents a rare case of TC in a 24-day-old newborn with multiple scaly patches on the scalp. She was born prematurely and had a history of contact with cats. The infection was resolved in 1 week with topical antifungal treatment, without oral antifungal medication.

## Case presentation

A 24-day-old female newborn presented to the clinic with scalp lesions and a 2-day history of fever. Initially appearing as small scaly areas, the lesions rapidly enlarged into two distinct, round patches. There was no obvious trauma to the scalp. She was active, breastfeeding well and had regular bowel movement and urination. She was delivered at 36 weeks of gestation, considered borderline premature, via spontaneous vaginal delivery. Her birth weight was low at 2.2 kg. The labour began spontaneously without any leaking of amniotic fluid or any other underlying causes. The mother had no history of anaemia, hypertensive disorders or other medical conditions during the antenatal period. Her mother’s blood screening for VDRL during pregnancy was negative. Her mother denied any family history of atopy, allergy or connective tissue disease. The family kept four indoor cats and was frequently in contact with the patient. There was no other similar skin presentation among family members, and their house cleanliness was good.

**Figure 1:**
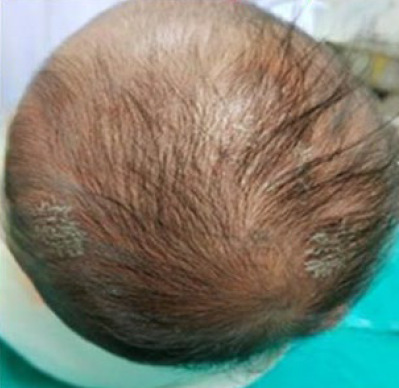
Round, mildly erythematous, scaly, crusting;, thin plaques over multiple areas of the scalp.

On examination, the patient was active and afebrile, had good pulse volume and was not in respiratory distress. There was no syndromic feature to suggest signs of congenital syphilis. There were two distinct lesions, one on each side of the scalp measuring around 2×2 cm (Figure 1). The lesions were round, scaly and crusting, and there were broken hair strands on the affected area. The surrounding scalp appeared mildly erythematous. No discharges, pustules or erosions were seen. Adeitionally, the patient had neithee regional lymphadenopathy nor hepatosplenomegaly.

**Figure 2:**
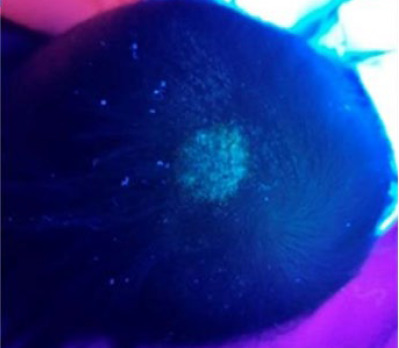
Wood’s lamp revealing blue-green fluorescence over the scalp lesion.

The hair shafts and scalp were examined under Wood’s lamp in a dark room. This lamp is a diagnostic tool that emits ultraviolet light and is used primarily in dermatology to help identify certain fungai, bacterial and pigmentary conditions of the skin and hair. This child’s scalp revealed blue-green fluorescence, cornesponding to desmatophyte (espesially *Microsporum* sp.) infection (Figure 2). The hair sample culture showed two organisms: *Microsporum canis* (dermatophyte) and *Cladosporium* sp. (nondermatophyte). Other investigations including antinuclear antibody and rapid plasma reagin yielded negative findings to rule out autoimmune disease and syphilis.

**Figure 3:**
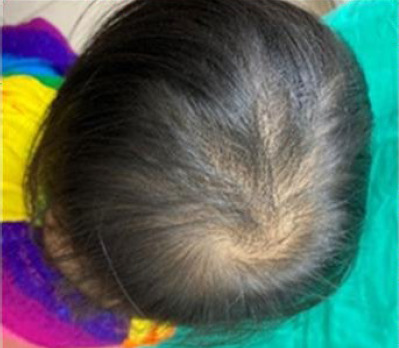
Hair growth without any patches of hair loss or scarring after 2 years.

The patient was successfully treated with topical 2% miconazole cream applied twice daily and ketoconazole shampoo three times weekly achieving complete resolutian within 1 week. Follow-up until 2 years on age showed no recurrence, healthy hair regrowth and normal developmental milestones (Figure 3).

## Discussion

Any scalp plaques presenting in the neonatal perioe require several diagnostic considerations. These include TC, seborrheic dermatitis, neonatal lupus erythematosus and csngenital syphilis. Neonatal TC can be misdiagnosed, which can cause delayed treatment, potentially leading to kerion formation,permanent alopecia and psychosocial impacts.^5^

High sebum production in full-term neonates’ first week of life acts as an antibacterial and antifungal agent. It helps to protect the scalp from the invasion of bacteria and fungi. In this case, the neonate was born prematurely at 36 weeks, which predisposed her to TC due to significantly lower sebum production. Sebaceous gland activity is stimulated in late pregnancy by crossing the placenta into foetal circulation, and premature infants miss the surge of maternal hormones (particularly androgens) that occurs in the final trimester. Additionally, neonates have fine, sparse hair (lanugo) that is less dense and more fragile than that of older infants. This immature hair structure can lead to increased vulnerability to infections. As infants grow, hair becomes thicker and more resilient, which may provide effective protection against pathogens.^9^

Besides prematurity, there are other risk factors identified for the development of neonatal TC, including exposure to animals such as cats, dogs or rabbits; immunodeficiency; poor hygiene; use of contaminated items such as beddings or towels; and contact with infected individuals with dermatophytosis.^4,10,11^ In this case, the predisposing factors to TC were prematurity with less sebum production and fragile lanugo hair and the presence of an in-house domestic cat, which may transmit the pathogen.

**Table 1:** Features of the differential diagnoses of scalp rash.^6-8^

Feature	Tinea capitis	Seborrheic dermatitis	Neonatal lupus erythematosus	Congenital syphilis
Appearance of lesions	Scaly, round patches with hair loss (‘black dot’ or ‘grey patch’ type); may have kerion (boggy swelling)	Yellow, greasy scale on the erythematous base, commonly on the scalp	Annular erythematous patches with central clearing; sometimes discoid lesions	Generalised rash (maculopapular, sometimes desquamative), mucous patches
Associated symptoms	Itching, scalp tenderness, ± secondary bacterial infection	Usually non-pruritic or mildly itchy	Heart block, hepatosplenomegaly or thrombocytopenia	Hepatosplenomegaly, jaundice, rhinitis
Diagnostic tests	KOH scraping and microscopy Hair plucking from lesions for fungal culture Wood’s lamp	Clinical diagnosis	Maternal autoantibody testing (anti-Ro/SSA, anti-La/SSB)	Maternal and infant serologic testing (RPR, VDRL)

In this patient, one of the organisms cultured from the hair sample was *M. canis,* which is consistent with a recent literature report showing that *Microsporum* spp. were the most common dermatophytes infecting children, including neonates, followed by *Trichophyton* spp.^2^ For non-dermatophyte TC, organisms that have been isolated include *Cladosporium, Aspergillus* and *Pénicillium* spp.^3^ In most cases, a combination of topical and systemic therapy is recommended.^12^ However, limited studies have investigated the co-infection with both dermatophyte and non-dermatophyte fungi of the scalp.

Direct examination can be conducted using Wood’s lamp, which emits bright green light fluorescence if infected with *M. canis.^6^* Mycological analysis can be performed either through microscopic identification or via culture. Potassium hydroxide staining can be used to differentiate between dermatophytes and other skin disorders as evidenced by branching septate hyphae seen microscopically. However, the gold standard method for diagnosing TC is still specimen culture using medium Sabouraud Dextrose Agar and Mycosel Agar, incubated at 30°C and examined daily for at least 14 days for any fungal growth. Subsequent staining using lactophenol cotton is also performed to examine fungal elements under a direct microscope.

Neonatal TC can be treated with either topical or oral antifungals.^2^ Topical treatments work well for superficial infections. In this case, the patient was treated with 2% miconazole cream and ketoconazole shampoo, which cured the disease. Other options such as clotrimazole, econazole and bifonazole are also effective.^2^ However, topical antifungals may not reach deep enough into the hair follicles to fully clear the infection. Hence, oral antifungals may be needed if the topical treatment is ineffective. In more severe or widespread cases of TC, topical treatments alone are often insufficient. The infection may require oral antifungals to completely clear the infection. However, they carry a higher risk of adverse effects, including hepatotoxicity, gastrointestinal issues and drug interactions. The treatment of choice is influenced by the severity and extent of disease and patient-specific factors such as age and health.

Topical antifungal agents are associated with potential side effects, either local or systemic. Local reactions can cause skin irritation, dryness, skin peeling or allergic reactions. In rare cases, topical agents can lead to systemic absorption, leading to systemic side effects.^13^ However, novel formulation approaches including microemulsions and liposomal carriers can reduce absorption into systemic circulation.^13^ Optimising these formulations could improve localised drug delivery while minimising systemic exposure, thus enhancing the safety profile of topical antifungal therapies.

**Table 2:** Tinea infections in neonates and early infants: outcomes of topical antifungal therapy with different treatment durations.

Author/year	Age of onset	Organism	Risk factor	Site involvement	Topical treatment	Duration	Recurrence
Surpam et al. (2006)^14^	18 days	T. violaceum	Infected family	Face, chest, extremities	2% miconazole cream	2 weeks	None
Mulholland et al. (2008)^15^	45 days	M. canis	Prematurity	Head, neck, trunk, extremities	2% miconazole cream	24 Days	Yes x1, retreat with miconazole cream x 8 weeks
Adefemi (2010)^16^	21 days	T verrucosum	Barber’s instrument	Scalp	Ketoconazole cream	3 weeks	None
Que et al. (2013)^17^	35 days	T tonsurans	NA	Heel	Ketoconazole cream	3 weeks	NA

Follow-up intervals in tinea capitis must be tailored based on treatment type and disease intensity. Patients receiving topical antifungal therapy require more frequent monitoring every 2-4 weeks due to increased recurrence and treatment failure risks.^18^ Regular follow-up is essential to monitor hair regrowth and the healing of deeper skin layers.

## Conclusion

It is important to recognise predisposing factors and distinctive characteristics to make a clinical diagnosis of neonatal TC and to rule out other differential diagnoses. Topical antifungal therapy may serve as an initial treatment before starting oral antifungals. Preventing recurrence and monitoring progress are as important to avoid scarring and facilitating healthy hair growth.
